# Case Report: Anti-glomerular basement membrane disease following COVID-19 infection

**DOI:** 10.3389/fneph.2025.1591512

**Published:** 2025-09-02

**Authors:** Justin David Tse, Jackson Wang, Adarsh Bhat, Rajib Kumar Gupta

**Affiliations:** ^1^ Internal Medicine Residency Program, Sutter Roseville Medical Center, Roseville, CA, United States; ^2^ Summit Nephrology, Roseville, CA, United States; ^3^ Department of Pathology, University of California, Davis, Sacramento, CA, United States

**Keywords:** ESRD, anti-glomerular basement membrane disease, plasmapheresis, acute kidney injury, COVID-19, renal biopsy, dialysis

## Abstract

Anti-glomerular basement membrane (anti-GBM) disease is a rare autoimmune disorder characterized by circulating autoantibodies targeting type IV collagen, leading to rapidly progressive glomerulonephritis. We report a case of a 44-year-old African American female with a history of hypertension who presented with acute kidney injury, hematuria, and shortness of breath. She tested positive for COVID-19 and received antiviral therapy; however, her renal function rapidly deteriorated, with serum creatinine rising from 3.4 to 10 mg/dL. Serologic testing ruled out common autoimmune conditions, but elevated CH50 levels suggested ongoing immune activation. Renal biopsy demonstrated diffuse necrotizing crescentic glomerulonephritis with linear IgG staining, consistent with anti-GBM disease. Despite aggressive therapy, including plasmapheresis, corticosteroids, and dialysis, renal recovery was not achieved. Immunosuppressive therapy was deferred in light of her active COVID-19 infection and the risk of immunosuppression-related complications. This case highlights a potential association between COVID-19 and anti-GBM disease, suggesting viral-induced endothelial injury and aberrant immune activation as possible mechanisms. Given emerging reports of autoimmune kidney diseases following COVID-19, further research is needed to clarify this relationship and guide optimal management. This is particularly important for patients who present with severe renal dysfunction in the context of an active infection.

## Introduction

COVID-19 has significantly reshaped our understanding of viral infections, revealing unexpected complications, including associations with autoimmune diseases. Anti-glomerular basement membrane (anti-GBM) disease is a rare autoimmune condition characterized by autoantibodies directed against type IV collagen, typically presenting as rapidly progressive glomerulonephritis. Emerging evidence suggests that viral infections such as COVID-19 may precipitate anti-GBM disease, possibly through mechanisms involving endothelial injury and aberrant immune activation. Here, we present a unique case of biopsy-proven anti-GBM disease following COVID-19 infection in a 44-year-old African-American female. This case emphasizes the importance of recognizing autoimmune complications in patients with COVID-19 presenting with acute kidney injury, even in the absence of significant pulmonary involvement.

## Case report

We present a 44-year-old African American female with a past medical history of hypertension on lisinopril 20mg. She had prior COVID-19 infection with subsequent two-dose COVID-19 vaccination series, who presented to the emergency department with a one-week history of shortness of breath, dark-colored urine, and generalized weakness. She was previously seen in the emergency department and was recommended to increase her hydration, given her dark-colored urine, and was sent home. Her creatinine was noted to be 1.2 mg/dL, which was around her baseline during this time. She was also treated for bronchitis earlier in the year and discharged with antibiotics but she stated that these therapies only marginally improved her symptoms.

**Figure 1:**
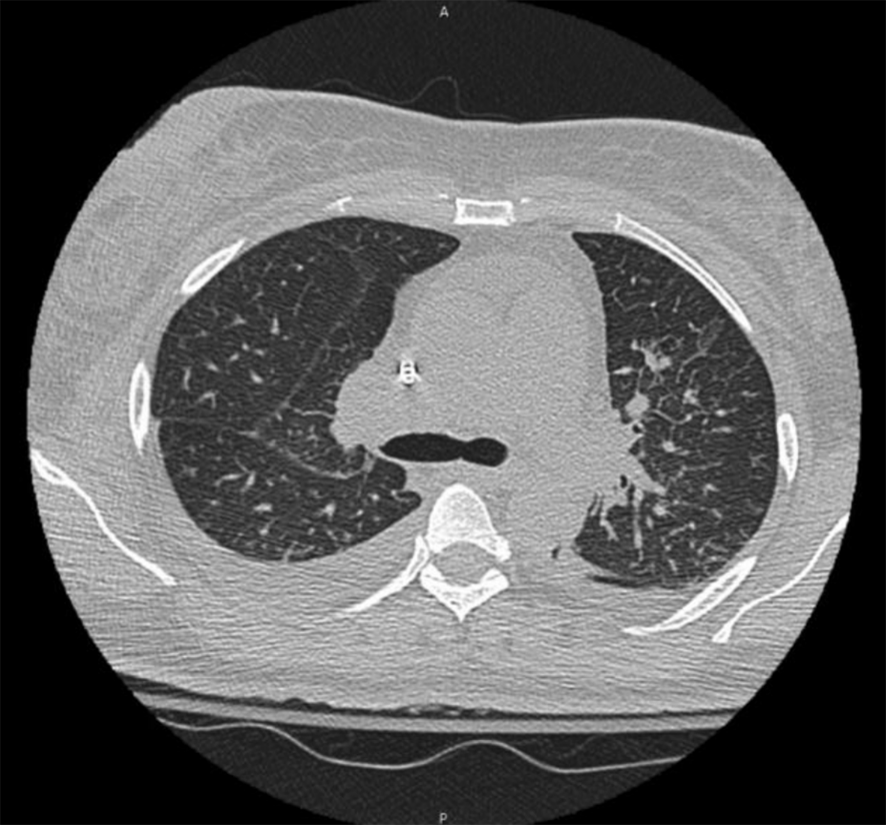
CT chest demonstrating a small right pleural effusion and moderate ground-glass opacities.

On the subsequent hospital admission, she reported worsening respiratory symptoms, and the inability to lift her arms above her head, which prompted her to call an ambulance. She denied any recent travel, sick contacts, or changes in medications. She denied any hemoptysis. Vital signs demonstrated tachycardia, with a heart rate of 105 bpm, blood pressure: 121/85 mmHg, and a respiratory rate of 20. Lab work was notable for sodium of 132 mmol/L, and a creatinine of 3.4 mg/dL, eGFR: 16mL/min, and CRP of 69 mg/L. She had an elevated procalcitonin of 4.90 ng/mL, albumin of 1.6 g/dL, and urine protein at 1288 mg/day. Urine sodium and urine creatinine were normal. Urinalysis demonstrated >100 RBCs and 3+ urine blood. Relevant laboratory values on admission are summarized in **Table 1**. CT chest was negative for any obvious infiltrate, aside from a small pleural effusion and moderate ground-glass opacities (**Figure 1**). Transthoracic echocardiography was normal, indicating no evidence of cardiac dysfunction or fluid overload during her acute illness. The elevated procalcitonin and CRP in combination with her prior incomplete response to outpatient antibiotics, raised concern for bacterial superinfection. Although blood and sputum cultures were negative, the presence of moderate ground-glass opacities on chest CT raised the possibility of an atypical or resolving pneumonia. Given these findings, the patient was empirically started on azithromycin and ceftriaxone for possible bacterial pneumonia complicating COVID-19. Antibiotics were continued during the acute phase of hospitalization given her initial presentation suggestive of sepsis, although no focal bacterial source was confirmed. Given her worsening respirations, she was admitted to the ICU for acute respiratory failure requiring supplemental oxygen but was subsequently downgraded the next day given improvement in her respirations.

**Table 1:** Relevant lab values on admission.

Complete Blood Count; ESR; Procalcitonin	Value	Reference Range
**WBC (K/uL)**	8.3	4.0-11
**RBC (M/uL)**	4.39	3.9-5.4
**Hemoglobin (g/dL)**	10.9	11.7-15.5
**Hematocrit (%)**	33.4	35-47
**MCV (fL)**	76	80-100
**MCH (pg)**	24.8	27-33
**MCHC (g/dL)**	32.6	31-36
**RDW (%)**	15.5	<16.4
**ESR (mm/hr)**	69	0-25
**Procalcitonin**	4.90	0-0.24
**Sodium (mmol/L)**	132	136-145
**Potassium (mmol/L)**	3.8	3.5-5.1
**Chloride (mmol/L)**	101	98-110
**Bicarbonate (mmol/L)**	25	21-32
**Anion Gap (mmol/L)**	6	1-12
**Glucose (mg/dL)**	86	70-100
**BUN (mg/dL)**	37	6-25
**Creatinine (mg/dL)**	3.40	0.40-1.00
**eGFR (mL/min)**	16	>60
**Albumin (g/dL)**	1.6	3.2-4.7
**ESR (mm/hr)**	69	0-25
**Procalcitonin**	4.90	0-0.24
**Hemoglobin (g/dL)**	10.9	11.7-15.5

**Figure 2:**
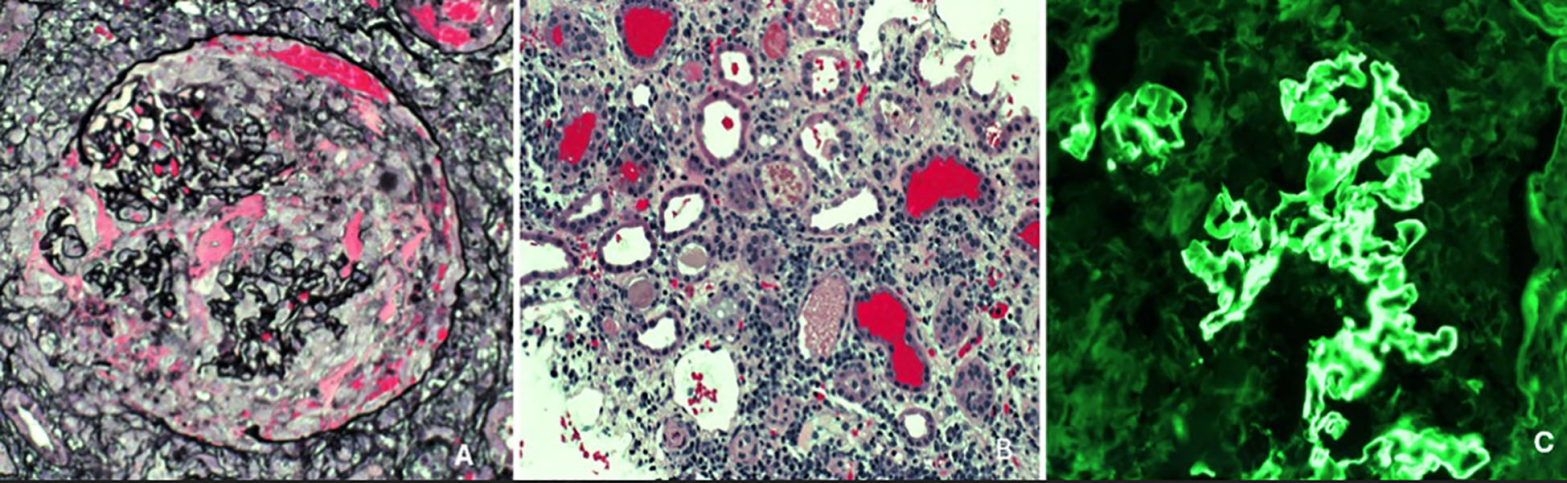
Jones methenamine silver (JMS) stain depicting a representative glomerulus showing capillary tuft rupture associated with necrosis and a cellular crescent (necrotizing crescentic GN), x400 [image **(A)**]; JMS stain showing numerous dilated tubules (acute tubular injury), interstitial inflammation and numerous red cell casts, x200 [image **(B)**]. Immunofluorescence stain for immunoglobulin IgG showing bright linear staining of the glomerular capillary loops (characteristic of anti-GBM disease), x200 [image **(C)**].

**Figure 3:**
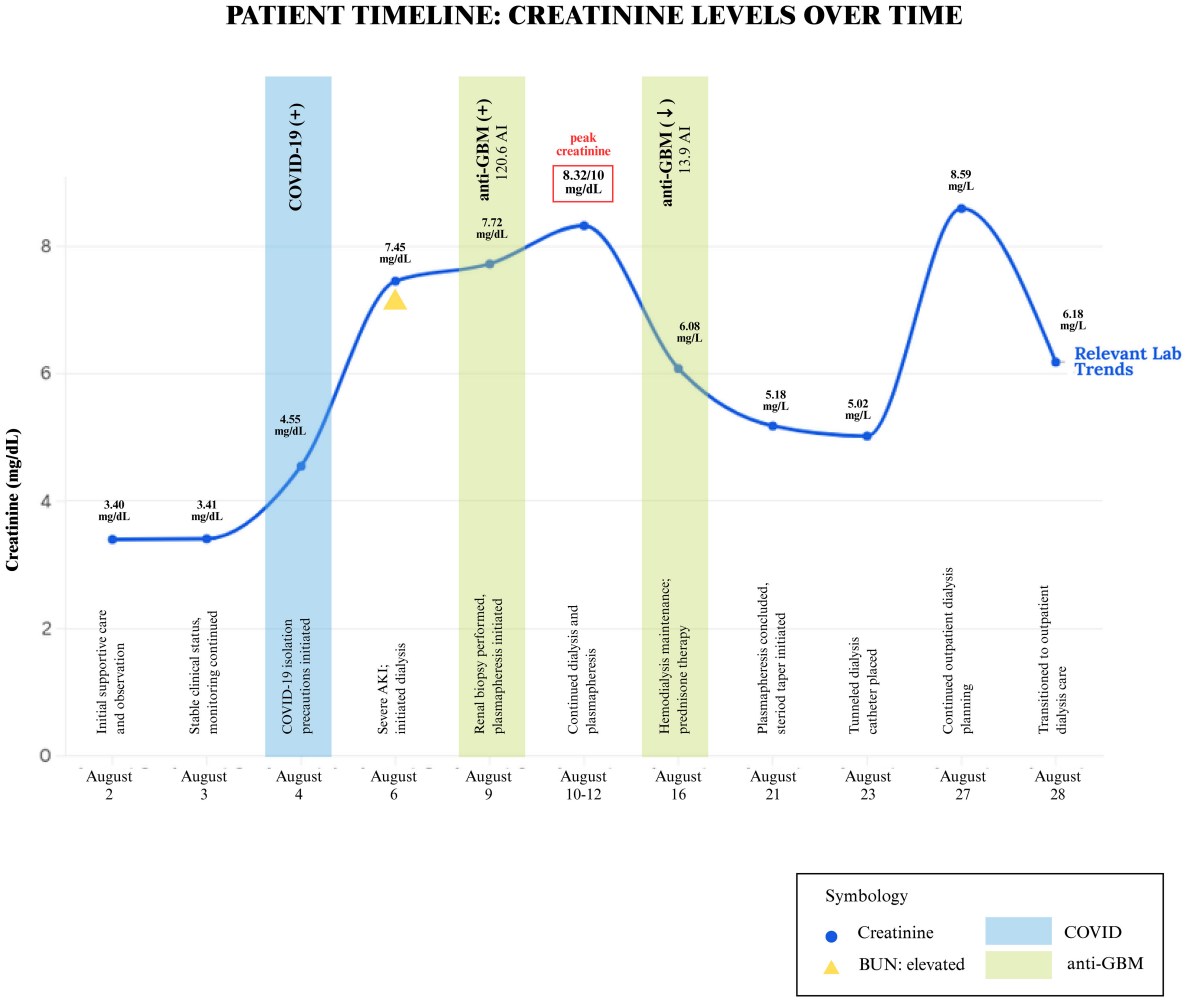
Timeline of relevant hospital events.

Despite being downgraded from the ICU, the patient’s creatinine continued to worsen, from 3.4 mg/dL to 10 mg/dL over the week. The patient was originally thought to be experiencing prerenal azotemia in the setting of dehydration; however, increased fluid administration and IV albumin did not improve her creatinine and overall renal function. There was initial concern for MIS-A in the setting of COVID-19, however, because there was only a single organ involvement, this diagnosis was deferred. In light of her young age and unexplained renal failure, additional serologic tests were performed to evaluate for autoimmune causes. All results were negative, including HIV, ANA, ANCA, dsDNA, acute hepatitis panel, and ASO. Complement C3 and C4 levels were normal, however, CH50 complement levels were >60 u/mL. She underwent a renal biopsy, which revealed findings of a diffuse necrotizing and crescentic glomerulonephritis (88% of glomeruli with extensive capillary tuft rupture, fibrinoid necrosis and cellular/fibrocellular crescents) along with tubulointerstitial edema, diffuse acute tubular injury, numerous red cell casts and bright linear IgG staining on immunofluorescence, consistent with the diagnosis of anti-GBM disease (**Figure 2**). Serologic testing supported the diagnosis, with an anti-GBM antibody titer of 120.6 AI. Given these findings, the patient underwent dialysis and began plasmapheresis and steroids. A timeline of the patient’s clinical course and renal function trends is shown in **Figure 3**. Dialysis was performed via intermittent hemodialysis, and plasmapheresis was conducted using centrifugal plasmapheresis with albumin replacement fluid and no specialized filters. Additional immunosuppressive agents (e.g., cyclophosphamide, rituximab) were considered but ultimately deferred due to her active COVID-19 infection and already severe kidney injury. She underwent multiple sessions of dialysis and seven rounds of plasmapheresis with improvement in anti-GBM Ab from 120.6 AI to 13.9 AI; however, there were no signs of renal recovery. She remained anuric, and her steroid taper was expedited. Additionally, she experienced episodes of anemia with a hemoglobin drop to 6.6 g/dL (baseline at approximately 8 g/dL) with thrombocytopenia of 50 K/uL. Further evaluation was completed but was unremarkable as her anemia improved throughout the hospitalization. This workup is detailed in **Supplementary Material**.

As she progressed through her hospital stay, her thrombocytopenia improved without intervention. Notably, the thrombocytopenia occurred at the peak of her illness when anti-GBM titers were highest and inflammatory markers were markedly elevated. It is also possible that plasmapheresis contributed to thrombocytopenia but deemed unlikely as these levels recovered as the antibody levels fell and the inflammatory response subsided. She is now receiving tri-weekly hemodialysis, requiring close follow-up by nephrology with tentative plans to transition to peritoneal dialysis and potential renal transplant.

## Discussion

This case illustrates the complexities involved in managing a patient with multiple comorbidities, including hypertension, acute kidney injury, sepsis, and COVID-19 pneumonia, all complicated by a diagnosis of anti-GBM disease. Anti-GBM disease is a rare but severe autoimmune, small vessel vasculitis disorder characterized by the presence of circulating autoantibodies that specifically target the glomerular basement membrane of the kidneys (specifically targeting the Goodpasture antigen, the NC1 domain of the α3 chain of type IV collagen in the basement membrane), and in some cases the alveolar basement membrane of the lungs as well. Anti-GBM disease is typically triggered by a combination of genetic predisposition as well as environmental factors such as infections or exposure to chemicals. Patients with anti-GBM disease often present with hematuria, proteinuria, and rapidly declining kidney function, and in cases involving the lungs, symptoms can sometimes include hemoptysis and shortness of breath. Diagnosis is confirmed via detection of positive circulating anti-GBM antibodies or a renal biopsy, as was found in our patient.

Anti-GBM disease typically occurs in Caucasian males with a bimodal age distribution, making our patient’s presentation unique (7, 8). There is emerging evidence that viral infections such as COVID-19 can trigger or exacerbate autoimmune responses leading to anti-GBM disease, although the mechanism is still unclear given the novelty of the virus (9–11). There has been a known 50% increase in the amount of anti-GBM disease associated with COVID-19, and a notable 3-fold increase from 2018-2022 (11, 12).

Compared to prior reports, our patient demonstrates several distinctive features. She is one of the few reported African American women with biopsy-confirmed anti-GBM disease following COVID-19. Uniquely, her case lacked any overt alveolar hemorrhage, and standard immunosuppressive therapy was not given. Despite a decline in antibody titers following plasmapheresis, she failed to recover renal function and remains dialysis-dependent. These findings highlight the challenges of managing autoimmune kidney disease in the setting of concurrent infection, where standard therapies may not be feasible. In our patient’s case, common autoimmune etiologies such as lupus were ruled out. CH50 remained >60 u/mL, suggesting ongoing immune activity and inflammation, leaving COVID-19 as a cause. A comparative summary of these reports is provided below in **Table 2**.

**Table 2:** Comparative summary of similar cases.

Citation	Demographics	Onset Relative to COVID-19	Pulmonary Involvement	Treatment	Outcome
**Sebastian et al. (2021) – Case 1** (1)	36F	~4 weeks post COVID-19	Yes – mild	Steroids, plasmapheresis, cyclophosphamide, dialysis	Partial Renal Recovery
**Sebastian et al. (2021) – Case 2** (1)	18M	~8 weeks post COVID-19	Yes – mild	Steroids, plasmapheresis, cyclophosphamide, dialysis	CKD, not dialysis dependent
**Sebastian et al. (2021) – Case 3** (1)	52M	~6 weeks post COVID-19	Yes – mild	Steroids, plasmapheresis, cyclophosphamide, dialysis	Dialysis-dependent
**Sebastian et al. (2021) – Case 4** (1)	32F	~6 weeks post COVID-19	Yes – mild	Steroids, plasmapheresis, cyclophosphamide, dialysis	Dialysis-dependent
**Winkler et al. (2021)** (2)	31F	Concurrent COVID-19 infection. Anti-GBM relapse occurred during active COVID-19	Yes – pulmonary hemorrhage	Steroids, plasmapheresis, rituximab	Dialysis-dependent
**Babu et al. (2022)** (3)	59M	~8 weeks post COVID-19	No	Steroids, cyclophosphamide	Renal recovery
**Shenoy et al. (2022)** (4)	57M	Concurrent with COVID-19	Yes – COVID pneumonia only	Steroids, plasmapheresis deferred; no cyclophosphamide	Dialysis-dependent
**Hu et al. (2025)** (5)	60F	~4 weeks post COVID-19	No	Steroids, plasmapheresis, cyclophosphamide	Improved (no dialysis)
**Prendecki et al. (2020) – Case 1** (6)	73F	~1 week post COVID-19	No	Steroids, plasmapheresis, rituximab, cyclophosphamide	Dialysis-dependent
**Prendecki et al. (2020) – Case 2** (6)	45F	~5 weeks post COVID-19	No	Steroids, plasmapheresis, rituximab, cyclophosphamide	CKD V
**Prendecki et al. (2020) – Case 3** (6)	73F	~1 week post COVID-19	No	Steroids, plasmapheresis, rituximab, cyclophosphamide	Dialysis-dependent
**Prendecki et al. (2020) – Case 4** (6)	27M	~7 weeks post COVID-19	No	None (in ESKD)	Dialysis-dependent
**Prendecki et al. (2020) – Case 5** (6)	63F	~3 weeks post COVID-19	No	Steroids, plasmapheresis, rituximab, cyclophosphamide	CKD V, not on dialysis
**Prendecki et al. (2020) – Case 6** (6)	72F	~2 weeks post COVID-19	No	Steroids, plasmapheresis, rituximab, cyclophosphamide	CKD IV
**Prendecki et al. (2020) – Case 7** (6)	34F	~8 weeks post COVID-19	No	Steroids, plasmapheresis, rituximab, cyclophosphamide	Renal recovery
**Prendecki et al. (2020) – Case 8** (6)	37F	~2 weeks post COVID-19	No	Steroids, plasmapheresis, rituximab, cyclophosphamide	Renal recovery
**Averages**	48 years old	3-4 weeks average onset	29% with pulmonary involvement	88% on Immunosuppressive Therapy	29% achieved renal recovery

It is known that COVID-19 infection can infect and damage endothelial cell membranes, specifically type IV collagen in alveolar and glomerular basement membranes, precipitating autoantibodies as it is known that these viral infections can precipitate aberrant adaptive immune responses. The α3 chain of type IV collagen of the NC1 domain (α3(IV)NC1) is released into circulation upon damage. Circulating plasma cells can react with the α3(IV)NC1, encouraging these autoreactive immune cells, and promoting further cellular damage (1, 2, 6, 13, 14). There is also research to suggest that the Omicron variant often spares the alveolar compartment (15). Given the timing of her illness and the relative lack of pulmonary involvement, her COVID-19 infection was most likely due to the Omicron variant of SARS-CoV-2​. This variant is known for lower respiratory virulence, which aligns with our patient’s absent hemoptysis and only subtle lung findings. A comparative summary of similar reported cases is presented in **Table 2**. Notably, some literature has documented cases of new-onset anti-GBM disease shortly after COVID-19 vaccination, suggesting a robust immune stimulus from either COVID-19 infection or vaccination may trigger anti-GBM autoimmunity in susceptible individuals (16). Although a clear causative link remains uncertain.

While collapsing glomerulopathy (COVAN) remains the most frequently reported COVID-related glomerular disease, anti-GBM disease is far less common, highlighting the clinical significance of our case. There have been several reports of new-onset anti-GBM disease occurring shortly after COVID-19 vaccination, suggesting that a robust immune stimulus from either infection or vaccination can trigger anti-GBM autoimmunity in susceptible individuals (13). Genetic predisposition also plays a role in anti-GBM disease, with a strong association seen with the HLA-DR15 board serotype, with nearly 80% of patients with anti-GBM disease carrying this variant (7). Additionally, individuals with high-risk APOL1 genotypes are more likely to develop COVID-19–associated collapsing glomerulopathy (COVAN), particularly among African Americans. However, to date, no link has been established between APOL1 variants and, anti-GBM disease, highlighting an area for future research. It can be speculated that APOL1 risk variants might exacerbate interferon-γ signaling and thereby contribute to endothelial injury (17, 18). APOL1 genotyping was not performed in our patient, as her biopsy findings were diagnostic of anti-GBM disease and lacked features of COVAN. HLA typing was also not performed during this hospitalization but is currently being evaluated during her transplant workup.

Immunosuppressive therapy was carefully considered when treating our patient with anti-GBM disease. However, given her clinical status and concerns for ongoing COVID pneumonia and declining renal function, immunosuppressive therapy was deferred given concerns for rapid decompensation. Our patient received seven sessions of plasmapheresis, which resulted in a marked decline in anti-GBM antibody titers (from 120.6 to 13.9 AI). However, despite this biochemical response, there was no evidence of renal recovery, likely reflecting late presentation and the need to delay immunosuppression due to concurrent COVID-19. She is now dialysis dependent, requiring close follow-up with nephrology with plans for renal transplantation.

Strengths of this case include its unique patient demographics, clear biopsy-proven diagnosis, and detailed clinical follow-up. Limitations include the inability to initiate immunosuppression promptly due to active COVID-19 infection, potentially influencing renal recovery. Key take-home messages from this case include: (1) clinicians should consider testing for anti-GBM antibodies in COVID-19 patients with unexplained rapidly progressive renal dysfunction; (2) renal biopsy remains critical for diagnosis; and (3) balancing immunosuppressive therapy risks in the setting of active infections requires multidisciplinary collaboration.

## Conclusion

This case underscores three important clinical considerations: First, clinicians must maintain high vigilance for anti-GBM disease in patients with COVID-19–associated acute kidney injury, particularly when patients present with hematuria, proteinuria, and progressive renal dysfunction, even if pulmonary involvement is absent. Second, rapid serologic testing and timely renal biopsy are essential steps for an accurate diagnosis and optimal patient management. Third, this case highlights the therapeutic complexity of initiating immunosuppressive therapy during active viral infections, emphasizing the need for personalized, multidisciplinary approaches that carefully balance risks and benefits. Future studies incorporating HLA typing and APOL1 genotyping may provide valuable insights into genetic susceptibility, particularly in African American populations. As more cases emerge, further data are needed to clarify the optimal timing of immunosuppression and the role of host genetic factors in infection-associated glomerulonephritis.

## Data Availability

The original contributions presented in the study are included in the article/**Supplementary Material**. Further inquiries can be directed to the corresponding author.
