# Supplementary figures and images for: Case Report: Anti-glomerular basement membrane disease following COVID-19 infection
Source: Front Nephrol. 2025 Sep 2;5:1591512. doi: 10.3389/fneph.2025.1591512 (PMC12436124; doi:10.3389/fneph.2025.1591512)

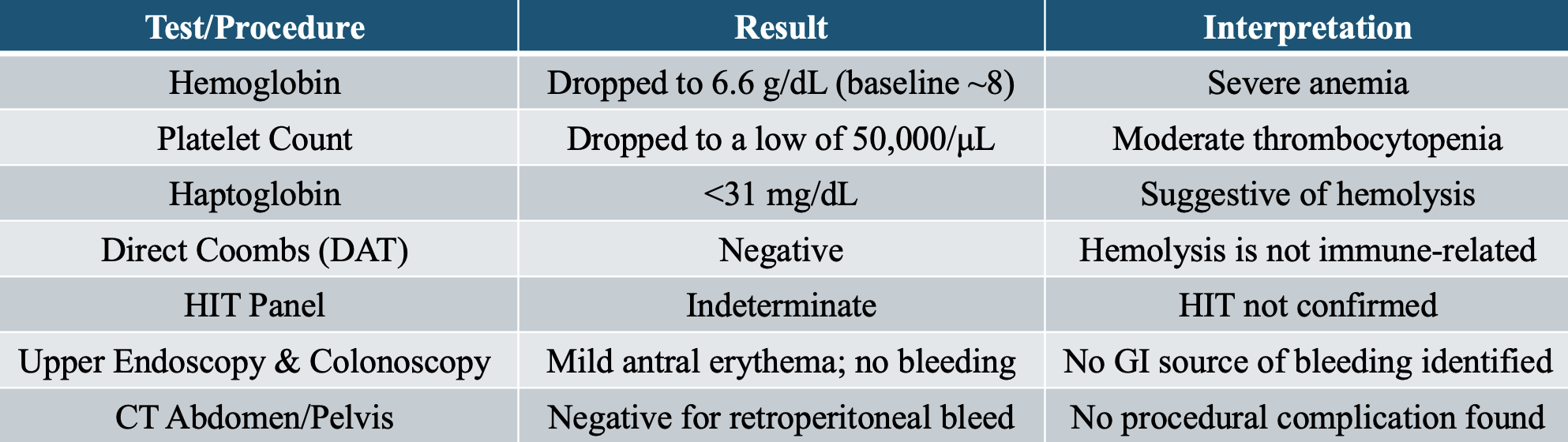

Supplement: Supplementary file 1 [file Image1.jpeg]

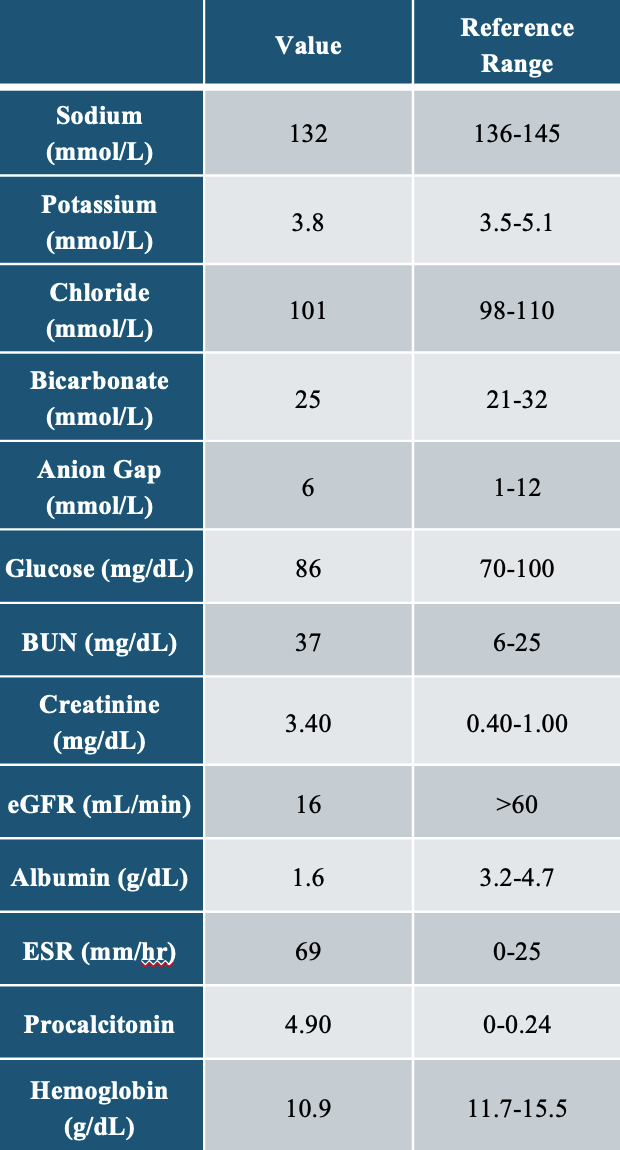

Supplement: Supplementary file 2 [file Image2.jpeg]

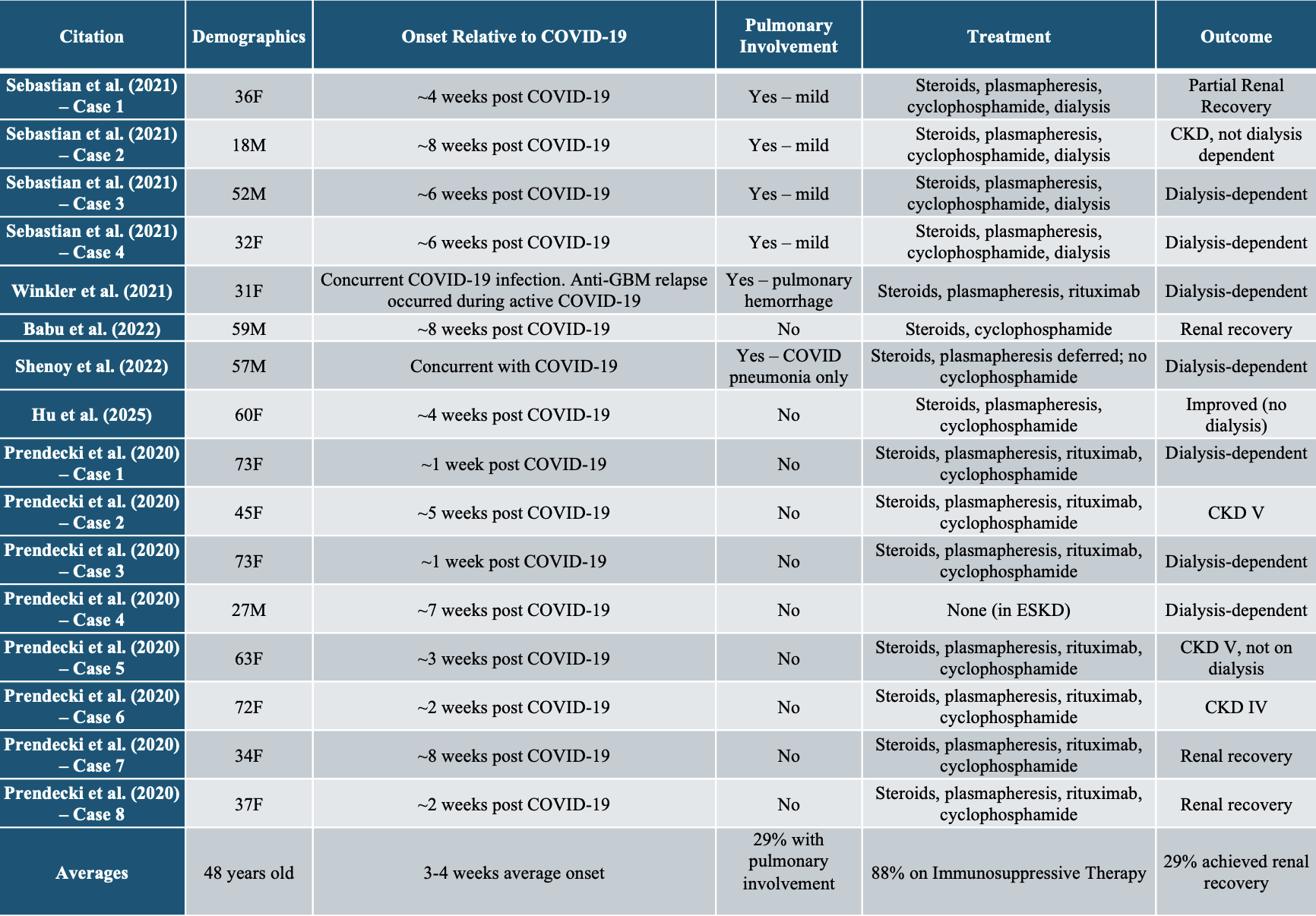

Supplement: Supplementary file 3 [file Image3.jpeg]
