# Supplementary material for: Case Report: Anti-glomerular basement membrane disease following COVID-19 infection
Source: Front Nephrol. 2025 Sep 2;5:1591512. doi: 10.3389/fneph.2025.1591512 (PMC12436124; doi:10.3389/fneph.2025.1591512)
Supplement: Supplementary file 4 [file Supplementaryfile1.pptx]

## Slide 1
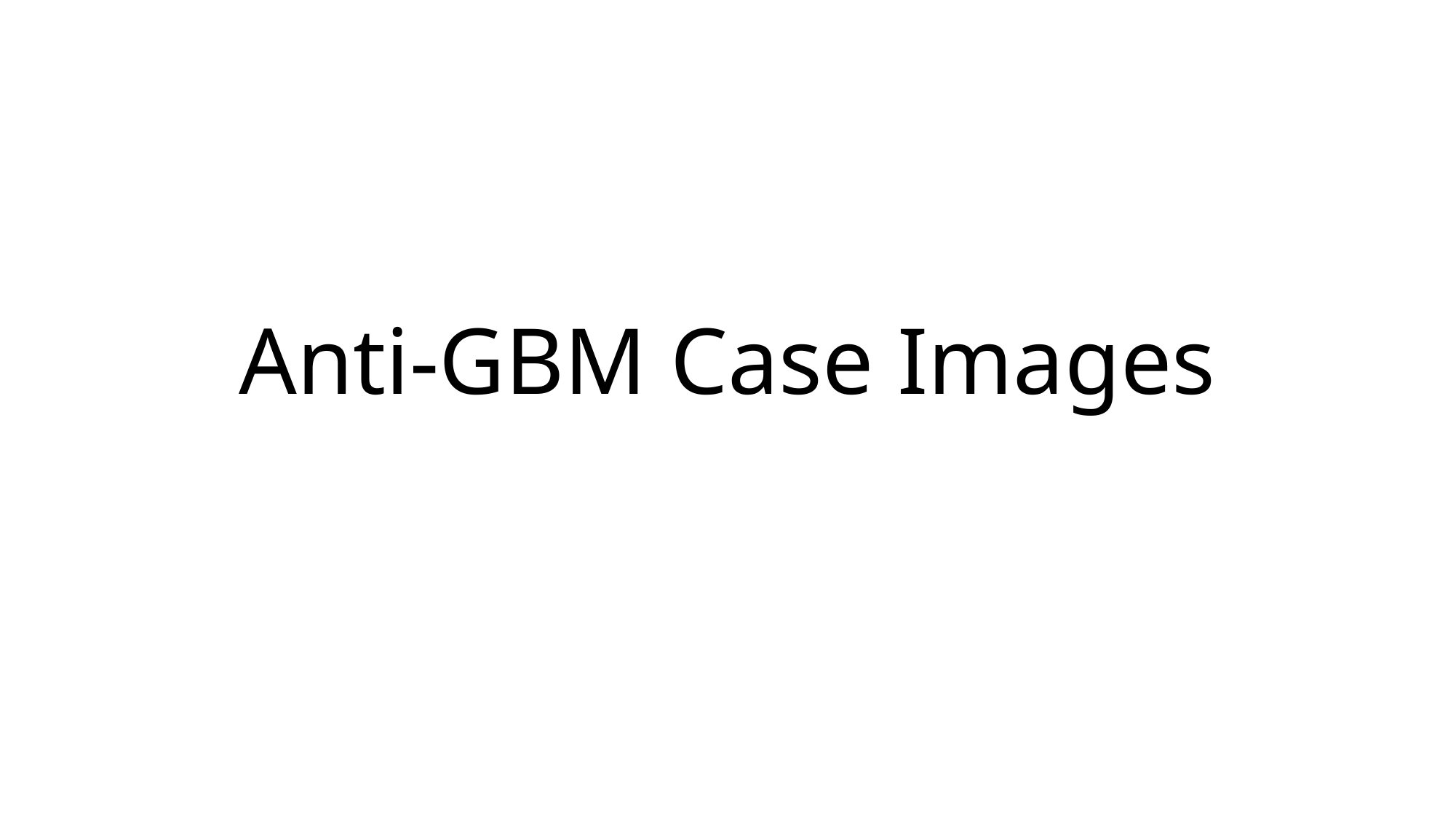

# Anti-GBM Case Images

## Slide 2
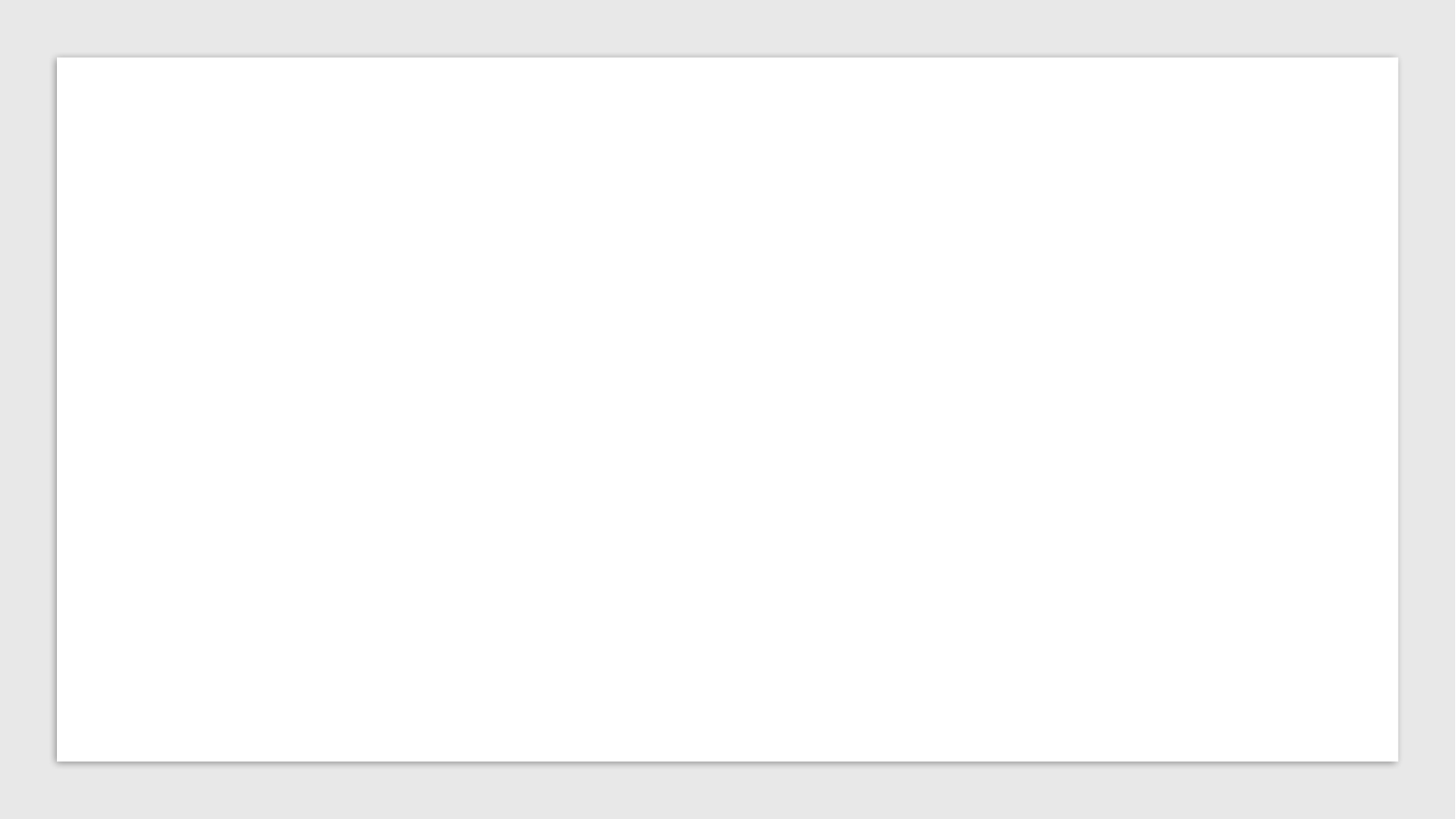

## Slide 3
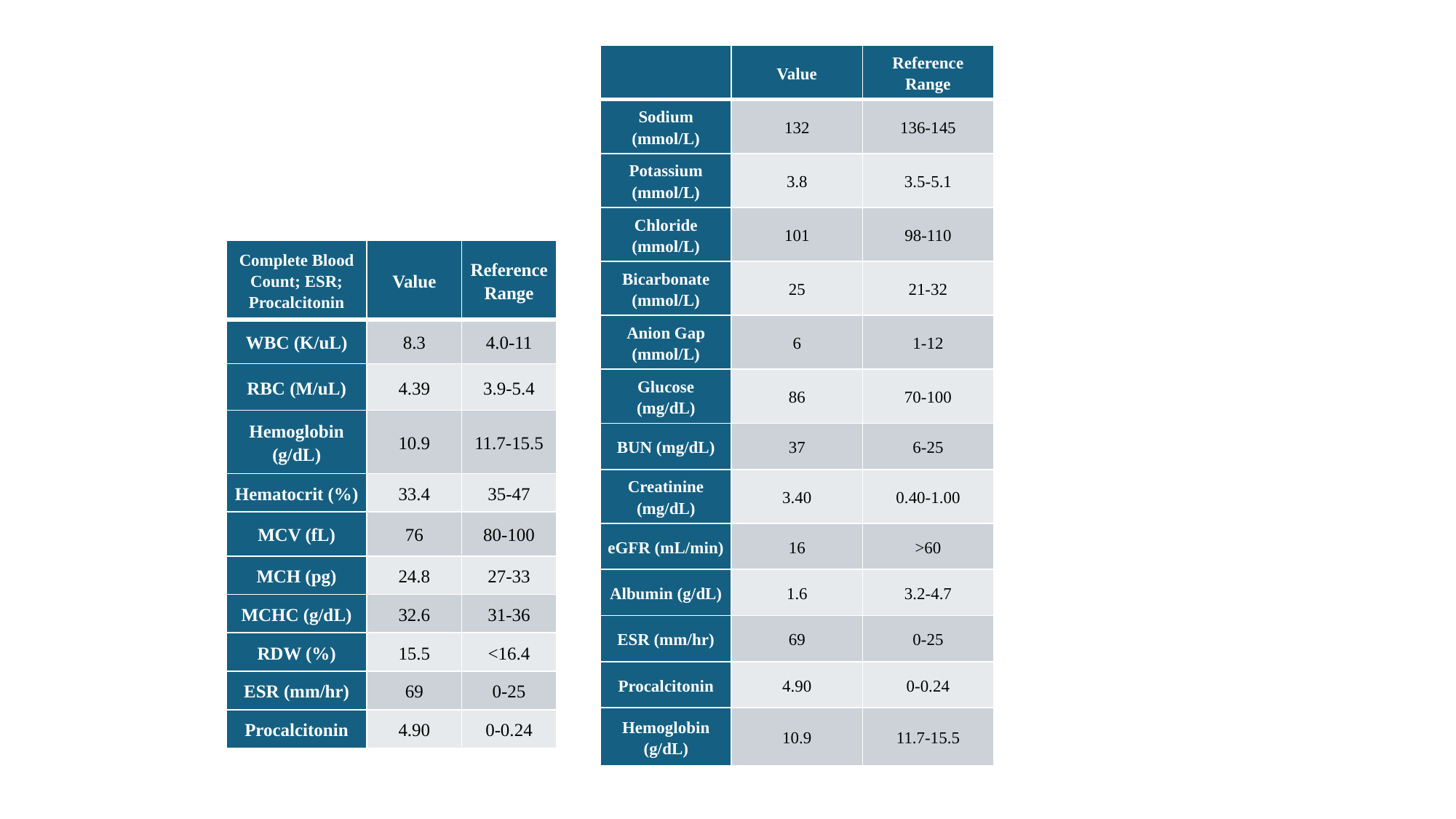

#
| | Value | Reference Range |
| --- | --- | --- |
| Sodium (mmol/L) | 132 | 136-145 |
| Potassium (mmol/L) | 3.8 | 3.5-5.1 |
| Chloride (mmol/L) | 101 | 98-110 |
| Bicarbonate (mmol/L) | 25 | 21-32 |
| Anion Gap (mmol/L) | 6 | 1-12 |
| Glucose (mg/dL) | 86 | 70-100 |
| BUN (mg/dL) | 37 | 6-25 |
| Creatinine (mg/dL) | 3.40 | 0.40-1.00 |
| eGFR (mL/min) | 16 | >60 |
| Albumin (g/dL) | 1.6 | 3.2-4.7 |
| ESR (mm/hr) | 69 | 0-25 |
| Procalcitonin | 4.90 | 0-0.24 |
| Hemoglobin (g/dL) | 10.9 | 11.7-15.5 |
| Complete Blood Count; ESR; Procalcitonin | Value | Reference Range |
| --- | --- | --- |
| WBC (K/uL) | 8.3 | 4.0-11 |
| RBC (M/uL) | 4.39 | 3.9-5.4 |
| Hemoglobin (g/dL) | 10.9 | 11.7-15.5 |
| Hematocrit (%) | 33.4 | 35-47 |
| MCV (fL) | 76 | 80-100 |
| MCH (pg) | 24.8 | 27-33 |
| MCHC (g/dL) | 32.6 | 31-36 |
| RDW (%) | 15.5 | <16.4 |
| ESR (mm/hr) | 69 | 0-25 |
| Procalcitonin | 4.90 | 0-0.24 |

## Slide 4
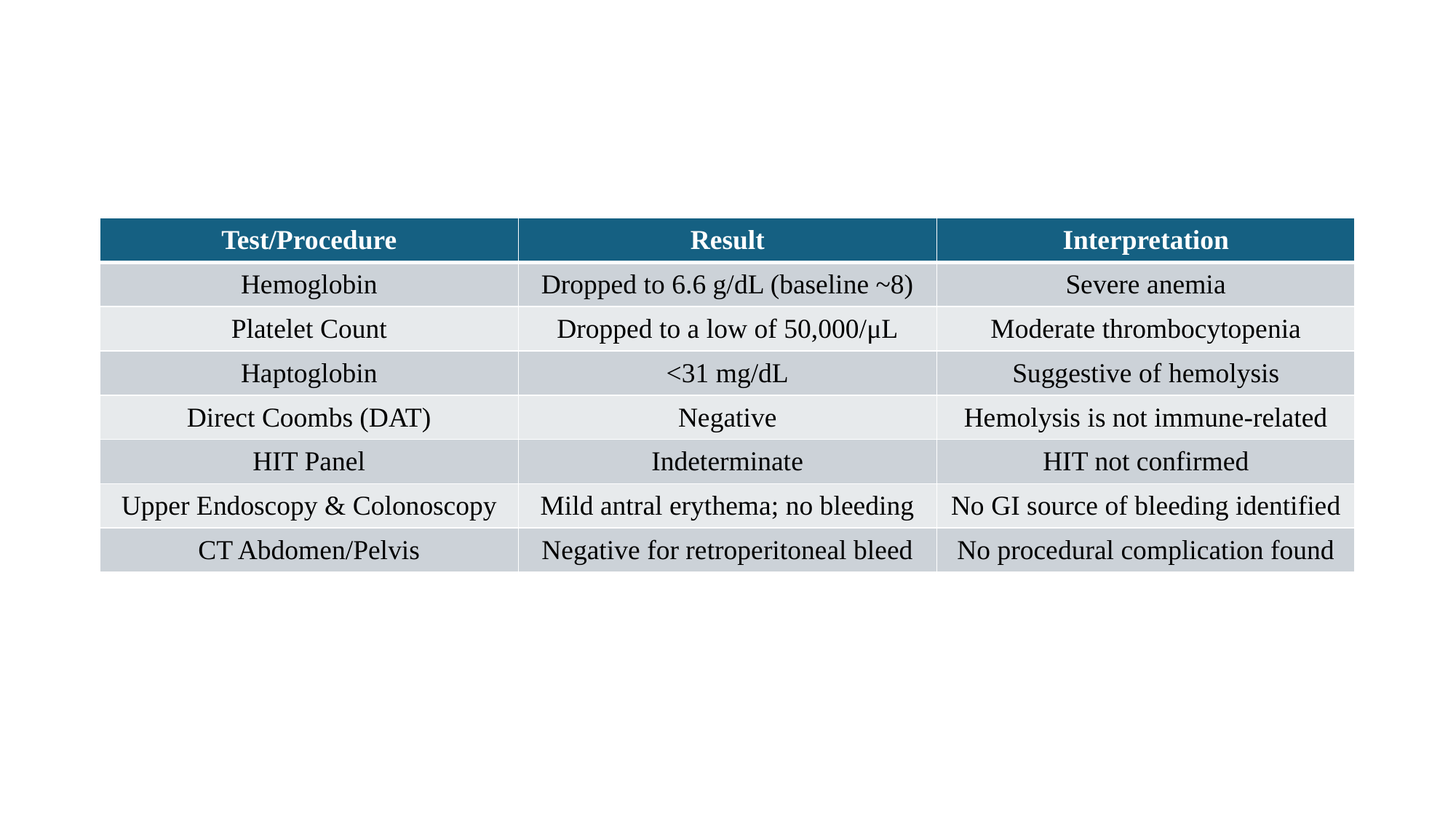

#
| Test/Procedure | Result | Interpretation |
| --- | --- | --- |
| Hemoglobin | Dropped to 6.6 g/dL (baseline ~8) | Severe anemia |
| Platelet Count | Dropped to a low of 50,000/μL | Moderate thrombocytopenia |
| Haptoglobin | <31 mg/dL | Suggestive of hemolysis |
| Direct Coombs (DAT) | Negative | Hemolysis is not immune-related |
| HIT Panel | Indeterminate | HIT not confirmed |
| Upper Endoscopy & Colonoscopy | Mild antral erythema; no bleeding | No GI source of bleeding identified |
| CT Abdomen/Pelvis | Negative for retroperitoneal bleed | No procedural complication found |

## Slide 5
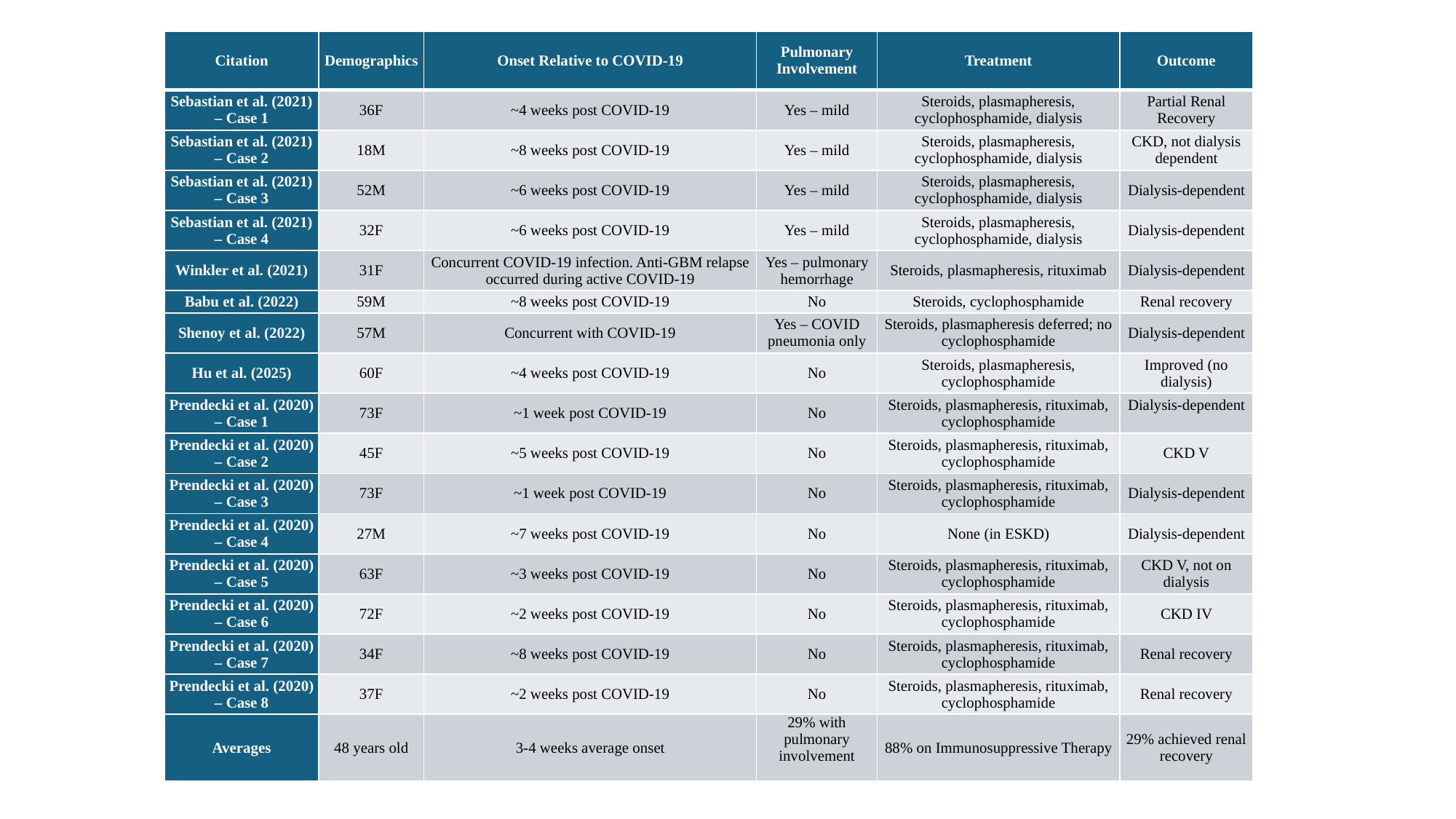

| Citation | Demographics | Onset Relative to COVID-19 | Pulmonary Involvement | Treatment | Outcome |
| --- | --- | --- | --- | --- | --- |
| Sebastian et al. (2021) – Case 1 | 36F | ~4 weeks post COVID-19 | Yes – mild | Steroids, plasmapheresis, cyclophosphamide, dialysis | Partial Renal Recovery |
| Sebastian et al. (2021) – Case 2 | 18M | ~8 weeks post COVID-19 | Yes – mild | Steroids, plasmapheresis, cyclophosphamide, dialysis | CKD, not dialysis dependent |
| Sebastian et al. (2021) – Case 3 | 52M | ~6 weeks post COVID-19 | Yes – mild | Steroids, plasmapheresis, cyclophosphamide, dialysis | Dialysis-dependent |
| Sebastian et al. (2021) – Case 4 | 32F | ~6 weeks post COVID-19 | Yes – mild | Steroids, plasmapheresis, cyclophosphamide, dialysis | Dialysis-dependent |
| Winkler et al. (2021) | 31F | Concurrent COVID-19 infection. Anti-GBM relapse occurred during active COVID-19 | Yes – pulmonary hemorrhage | Steroids, plasmapheresis, rituximab | Dialysis-dependent |
| Babu et al. (2022) | 59M | ~8 weeks post COVID-19 | No | Steroids, cyclophosphamide | Renal recovery |
| Shenoy et al. (2022) | 57M | Concurrent with COVID-19 | Yes – COVID pneumonia only | Steroids, plasmapheresis deferred; no cyclophosphamide | Dialysis-dependent |
| Hu et al. (2025) | 60F | ~4 weeks post COVID-19 | No | Steroids, plasmapheresis, cyclophosphamide | Improved (no dialysis) |
| Prendecki et al. (2020) – Case 1 | 73F | ~1 week post COVID-19 | No | Steroids, plasmapheresis, rituximab, cyclophosphamide | Dialysis-dependent |
| Prendecki et al. (2020) – Case 2 | 45F | ~5 weeks post COVID-19 | No | Steroids, plasmapheresis, rituximab, cyclophosphamide | CKD V |
| Prendecki et al. (2020) – Case 3 | 73F | ~1 week post COVID-19 | No | Steroids, plasmapheresis, rituximab, cyclophosphamide | Dialysis-dependent |
| Prendecki et al. (2020) – Case 4 | 27M | ~7 weeks post COVID-19 | No | None (in ESKD) | Dialysis-dependent |
| Prendecki et al. (2020) – Case 5 | 63F | ~3 weeks post COVID-19 | No | Steroids, plasmapheresis, rituximab, cyclophosphamide | CKD V, not on dialysis |
| Prendecki et al. (2020) – Case 6 | 72F | ~2 weeks post COVID-19 | No | Steroids, plasmapheresis, rituximab, cyclophosphamide | CKD IV |
| Prendecki et al. (2020) – Case 7 | 34F | ~8 weeks post COVID-19 | No | Steroids, plasmapheresis, rituximab, cyclophosphamide | Renal recovery |
| Prendecki et al. (2020) – Case 8 | 37F | ~2 weeks post COVID-19 | No | Steroids, plasmapheresis, rituximab, cyclophosphamide | Renal recovery |
| Averages | 48 years old | 3-4 weeks average onset | 29% with pulmonary involvement | 88% on Immunosuppressive Therapy | 29% achieved renal recovery |

## Slide 6
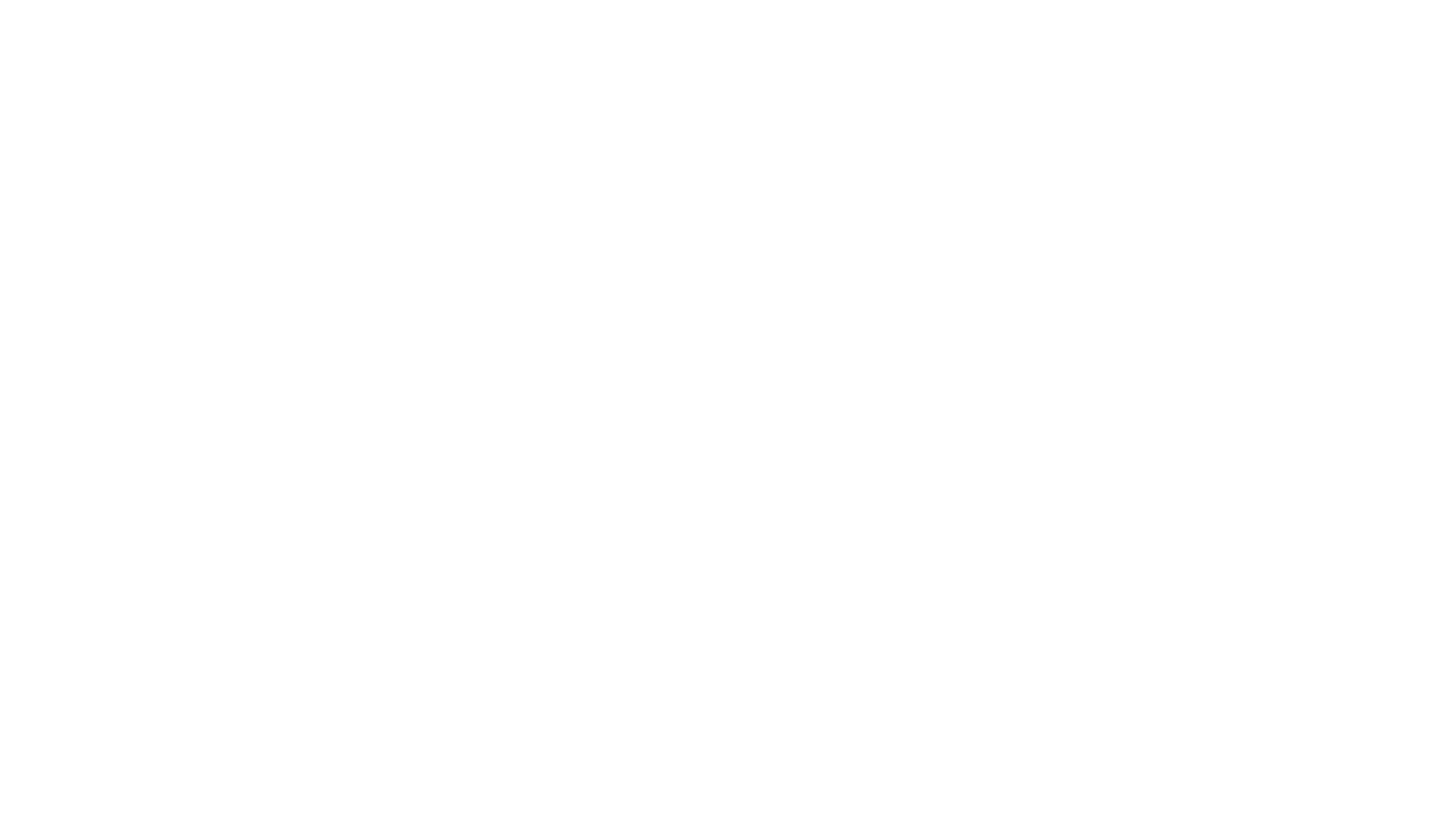

#
